# Radiation-associated bioprosthetic valve dysfunction: an initial case-control analysis

**DOI:** 10.3389/fcvm.2026.1783103

**Published:** 2026-06-19

**Authors:** Ioana Petrescu, Atif M. Islam, Brian Blair, Bibhu D. Mohanty

**Affiliations:** 1 Department of Cardiology, West Virginia University, Morgantown, WV, United States; 2 Morsani College of Medicine, University of South Florida Morsani College of Medicine, Tampa, FL, United States; 3 Department of Internal Medicine, University of South Florida Morsani College of Medicine, Tampa, FL, United States; 4 Division of Cardiovascular Sciences, University of South Florida Morsani College of Medicine, Tampa, FL, United States

**Keywords:** bioprosthetic valve, cardiotoxicity, hemodynamics, radiation-associated heart disease, radiotherapy, transvalvular gradient, valve durability, valvular heart disease

## Abstract

**Introduction:**

Radiation therapy (RT) for intrathoracic and breast malignancies has been associated with long-term valvular heart disease (VHD) in native valves. However, its impact on bioprosthetic heart valves remains unclear. We aimed to assess changes in transvalvular gradients following RT in patients with previously implanted bioprosthetic valves.

**Methods:**

We identified 23 patients who underwent bioprosthetic valve implantation followed by chest RT and compared them with matched controls who did not receive RT. Serial echocardiographic gradients were analyzed post-implantation, pre-RT, and at two intervals post-RT (within 12 months and at 12–24 months). Univariable linear regression was used to evaluate associations between RT dose metrics, timing of RT, and changes in transvalvular gradients.

**Results:**

Transvalvular gradients remained stable in control patients but increased significantly over time in the RT group (Friedman's test *P* < 0.0001). Both total RT dose and mean heart RT dose significantly predicted increases in transvalvular gradients within 12 months (R² = 0.41, *P* = 0.001) and between 12 and 24 months (R² = 0.62, *P* < 0.0001) of RT completion. Shorter time between valve implantation and RT also predicted greater gradient increases. No patients required re-intervention during the follow-up period.

**Conclusion:**

Chest RT is associated with early, dose-dependent elevations in transvalvular gradients among patients with bioprosthetic valves. Although the observed changes were modest and did not necessitate re-intervention, these findings highlight the need for long-term surveillance and further research to understand the durability of irradiated bioprosthetic valves.

## Introduction

As advancements in Cardiology and Oncology have led to marked improvement in patient survival, conditions found at the inter-disciplinary intersection have received greater attention (1). With recent advances in the procedural treatment of valvular heart disease (VHD), the number of valve replacements in the United States is projected to exceed 120,000 annually by 2026 (2, 3). The median age of prosthetic valve implantation (72 ± 5 years for surgical replacements and 81 ± 5 years for transcatheter replacements) overlaps with that of incident breast (62 ± 10 years) and lung cancer (70 ± 7 years) diagnoses (2, 4–7). As such, it becomes inevitable that recipients of prosthetic valves will also be diagnosed with cancer and, for loco-regional thoracic disease, radiation therapy to the chest will be an important component of the treatment strategy.

While previous research has focused on the now well-established deleterious effects of radiation therapy (RT) on native heart valves, the impact of RT on bioprosthetic valve prostheses remains largely unexplored (8–10). Chest RT causes diffuse interstitial fibrosis and collagen deposition in the heart which can result in a variety of cardiovascular complications including valvular heart disease manifesting as regurgitation, stenosis or both (11).

In the current analysis, we investigated the impact of RT (delivered total and median heart doses) on bioprosthetic valvular function over time.

## Materials and methods

### Study population

We retrospectively identified all patients who had bioprosthetic valve implantation between January 2015 and December 2021 with at least one post-implantation echocardiogram to define the cohort with available longitudinal follow up data. From this group, we selected index patients who had received RT to the chest, as well as controls in a 1:1 ratio matched for decade of age, sex, location of the bioprosthesis, and availability of echocardiographic studies within 3-month periods relative to valve implantation and timing of RT. For controls, we considered echocardiograms at equivalent time points in relation to the RT date of the respective index patient. Subjects with incomplete echocardiographic data were excluded. We performed direct chart review to obtain data regarding primary malignancy, total RT dose, mean heart RT dose, and transvalvular gradient values.

### Statistical analysis

Categorical variables are reported as counts (percentages). Numerical variables are reported as either mean (sd = standard deviation) if normal or approximately normal variables or median (range) for all other numerical variables. To assess normality of numerical variables, we used the Lilliefors-Kolmogorov–Smirnov. For comparisons by groups we performed Chi-square tests (or Fisher tests if cell count less than 5). We used the Kruskal–Wallis test to compare distributions of non-normal numerical variables. To analyze gradient changes over time, we employed Friedman's non-parametric test which is designed to explore non-normally distributed repeated-measures data. For a positive test, we followed up with *post hoc* pair-wise analysis using the Wilcoxon Signed-Rank Test with a Holm's correction for multiple comparisons. All *p*-values were two-sided and a *P-*value < 0.05 was considered to indicate statistical significance. Statistical analysis was performed using JMP (software version 17.0.0) and R statistical software (version 4.2.2).

## Results

We identified 23 index patients who had bioprosthesis implantation and RT during the study period. Characteristics of the 23 index patients and their matched controls are presented in Table 1. Most patients had replacement at the aortic position [15 (65%)]. Lung and breast primary malignancies accounted for most cases with an equal distribution (*N* = 11 for each); one patient had lymphoma involving the anterior mediastinum. The median total RT dose was 46 Gy (range, 30–60 Gy) (Table 2). The median age at implantation was 69 years (IQR, 62–77 years; range, 30–82 years) for the RT patients. The median age at RT was 73 years (IQR, 65–80 years; range, 31–88 years). The median time from valve implantation to RT was 33 months (IQR, 20–44 months; range, 3–81 months).

**Table 1:** Characteristics of overall population (*N* = 46).

Variables	RT status	
	Chest RT (*N* = 23)	No RT (*N* = 23)
Age (years) at implantation	69 (30–82)	67 (29–82)
Male Sex	11 (48%)	11 (48%)
Valve location		
Aortic	15 (65%)	15 (65%)
Mitral	6 (26%)	6 (26%)
Tricuspid	2 (9%)	2 (9%)
Valve Size		
23 mm	5 (22%)	5 (22%)
25 mm	6 (26%)	6 (26%)
27 mm	10 (43%)	10 (43%)
29 mm	2 (9%)	2 (9%)

Numbers represent *N* (%). Age at implantation is reported as median (range). RT, radiation therapy.

**Table 2:** Characteristics of chest RT patients (*N* = 23).

Primary malignancy	*N* (%)
Lung	11 (48%)
Breast	11 (48%)
Lymphoma	1 (4%)
Total RT dose (Gy)	46 (30–60)
Mean heart RT dose (Gy)	14 (6–27)
Time (months) from implantation to RT	33 (1–81)

Numbers represent *N* (%). Total RT dose, mean heart RT dose, time from implantation to RT are reported as median (range). RT, radiation therapy.

The baseline transvalvular gradient (measured within 1–3 months post-implantation) was similar between groups [median 8 mm Hg in the RT group [IQR, 4–10 mm Hg] vs. 8 in the control group [IQR, 4–12]; *P*-value=0.73], as was the gradient measured in the 6 months preceding RT or equivalent time point for controls [median 8 mm Hg in the RT group [IQR, 4–11 mm Hg] vs. 7 mm Hg in the control group [IQR, 3–11 mm Hg]; *P*-value = 0.83].

**Figure 1:**
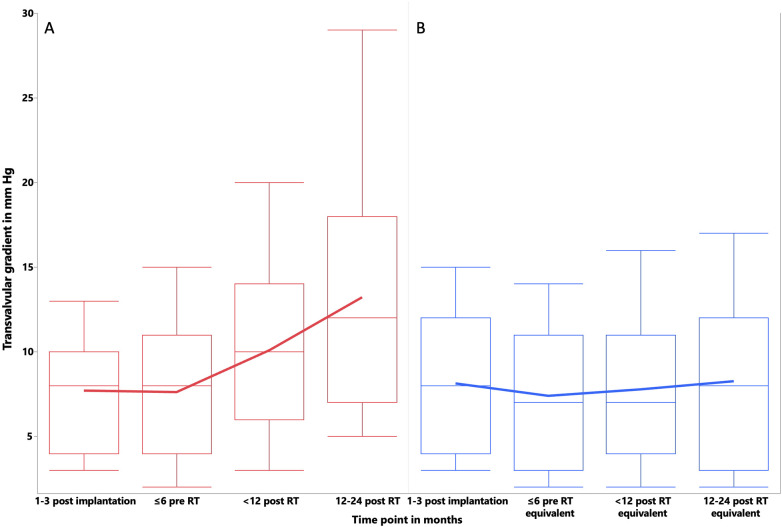
Changes in transvalvular gradient over time in **(A)** patients who received chest RT and **(B)** patients who did not receive RT. RT, radiation therapy.

We observed a steeper increase in the transvalvular gradient within the initial 12 months following RT and in the subsequent follow-up period for index patients compared to controls (Figure 1). The change in transvalvular gradient was significantly different in the RT group over time (Friedman's test *P*-value < 0.0001), but not in the control group (Friedman's test *P*-value = 0.26). Pair-wise comparisons demonstrated that gradients measured between 12 and 24 months [median 12 mm Hg (IQR, 7–18)] following RT were significantly different compared to post-implantation (median difference 4 mm Hg; median *P*-value = 0.0025) and pre RT (median difference 4 mm Hg; *P*-value = 0.0025) baseline values. The gradient measured within the first 12 months following RT [median 10 mm Hg (IQR, 6–14)] also differed from the post-implantation (median difference 2 mm Hg) and pre RT baselines (median difference 2 mm Hg), but failed to reach statistical significance (*P*-value = 0.08).

**Figure 2:**
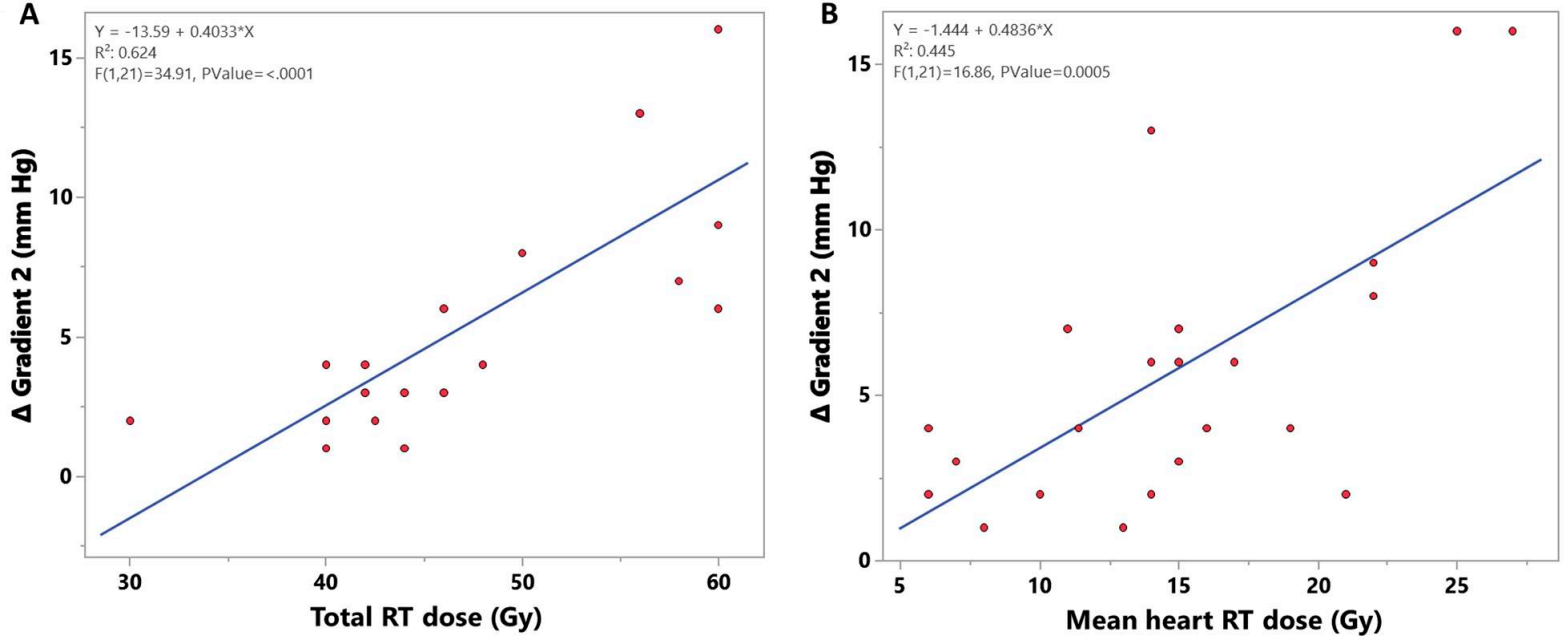
Univariable regression plots showing the effect of total RT dose **(A)** and mean RT heart dose **(B)** on the transvalvular gradient difference between the value calculated at 3–6 months pre RT and at > 12 months post RT (Δ gradient 2). RT, radiation therapy.

We used univariable linear regression to test if the total or median heart RT dose significantly predicted the change in gradient from the pre RT baseline to the gradient measured within 12 months (Δ Gradient 1) and between 12 and 24 months of RT completion (Δ Gradient 2). Analyses are presented in Table 3. For the total RT dose, the overall regression was statistically significant for both Δ Gradient 1 [β = 0.14; R^2^ = 0.41, F(1, 21) = 15, *P-*value = 0.001] and Δ Gradient 2 [β = 0.40; R^2^ = 0.62, F(1, 21) = 35, *P-*value < 0.0001]. In both cases, increasing RT doses significantly predicted larger Δ Gradient 1 and Δ Gradient 2. Mean heart RT dose behaved similarly in univariate regression models (Figure 2) and significantly predicted Δ Gradient 1 [β = 0.14; R^2^ = 0.41, F(1, 21) = 14, *P-*value = 0.001] and Δ Gradient 2 [β = 0.48; R^2^ = 0.45, F(1, 21) = 17, *P-*value = 0.0005].

**Table 3:** Univariable linear regression models.

Variables	β coefficient	R2	F statistic	*P*-value
Δ Gradient 1 as outcome				
Total RT dose	0.14	0.41	F(1,21) = 14	0.001
Mean heart RT dose	0.20	0.41	F(1,21) = 14.8	0.0009
Δ Gradient 2 as outcome				
Total RT dose	0.40	0.62	F(1,21) = 34.9	<0.0001
Mean heart RT dose	0.48	0.45	F(1,21) = 16.9	0.0005

Δ Gradient 1 is defined as the difference between the transvalvular gradient measured within 12 months of RT completion and the pre RT value. Δ Gradient 2 is defined as the difference between the transvalvular gradient measured after 12 months of RT completion and the pre RT value. RT, radiation therapy.

We also employed linear regression to explore the predictive ability of the time from implantation to RT on these gradient changes. Shorter intervals between implantation and RT predicted larger differences for both Δ Gradient 1 [β = −0.04; R^2^ = 0.21, F(1, 21) = 5.4, *P-*value = 0.03] and Δ Gradient 2 [β = −0.11; R^2^ = 0.35, F(1, 21) = 11.3, *P-*value = 0.003].

## Discussion

We investigated 23 valvular bioprosthetic recipients who also received post-implantation chest RT for intrathoracic or breast malignancy. In comparison to matched controls, we found a significant trend towards increasing transvalvular gradients over the subsequent 24 months, whereas no such trend was observed in controls over an equivalent period. We also found that total RT dose and mean heart RT dose significantly predict increases in transvalvular gradient changes over time with greater effect size.

These observations are consistent with prior data reporting the effect of RT on native cardiac valves. For survivors of childhood cancers treated with chest RT, there is a direct relationship between the mean heart dose and the rate of developing native VHD (12, 13). To our knowledge, this is the first analysis to explore the impact of RT dose on bioprosthetic heart valves.

Although the trends we observed are statistically significant, the association is relatively weak and the magnitude of change over the short observation period is clinically modest. Notably, no patients required valve re-intervention in our cohort. This finding is not surprising, as the latency of RT-associated native VHD is estimated to be 10–20 years (13–15), whereas our patients were followed over only 24 months following RT. Additionally, studies exploring the relationship between mean heart RT dose and the development of VHD date used older RT techniques. With contemporary RT planning, treatment fields are more conformal and increasingly focus on the target organ, reducing incidental exposure to cardiac structures. As a result, mean heart RT dose is expected to result in weaker relationships to VHD compared to the mean dose delivered to specific cardiac substructures (16). We did not have data available for the RT dose delivered to the respective bioprosthetic valve and our reliance on mean heart RT dose may also explain the relatively weak predictive ability.

If the latency of bioprosthetic degeneration is analogous to that of the development of native RT-associated VHD, long term follow-up would be required to capture the true extent of the RT effect on the bioprosthetic valves. However, bioprostheses have a documented life span of 10–20 years, and the cause of structural degeneration may be difficult to pinpoint (17). The rate of progression of RT-associated aortic stenosis appears similar for aortic stenosis arising in controls; however, patients with RT-associated native VHD are more symptomatic and are, consequently, referred earlier for valve replacement (18). Despite earlier intervention, patients with RT-associated VHD experience excess mortality compared to matched controls (18). Such patients are more likely to have mediastinal fibrosis, reduced preoperative ejection fraction, constrictive pericarditis, and a non-clampable porcelain aorta, contributing to technical challenges and longer cardiopulmonary bypass time (19). As a consequence, multiple studies have reported an elevated perioperative risk of complications and mortality after cardiac surgery for patients with prior chest RT (19–22). Novel, less invasive alternatives, such as transcatheter aortic valve replacement and transcatheter edge-to-edge repair of the mitral valve appear safe and effective in RT-associated VHD (19, 23–29) and direct comparison of surgical and transcatheter approaches have demonstrated the increased safety of the latter (21, 30).

As this analysis is an initial assessment of RT-effects on prosthetic valves, future studies may focus on longer follow-up to determine the true natural history of irradiated bioprosthetic valves and may consider how additional patient factors such as the development of cardiomyopathy and the receipt of chemotherapy may impact valve prognosis.

### Limitations

Our study has several important limitations. First, owing to its retrospective nature, it can only report association; we cannot comment on causality. In relying on chart review, data that may have been important to include in the analysis such as the emergence of patient-reported cardiac symptoms was not categorically collected. Second, as stated above, the number of patients was small, and the follow-up was short, which limits the statistical strength. The limited follow-up of 24 months was also likely too brief to detect clinically meaningful valve degeneration or other late manifestations of radiation-associated valve injury.

## Conclusion

In this matched cohort study, we observed a significant association between chest radiation-therapy and accelerated changes in transvalvular gradients among patients with bioprosthetic heart valves. While the magnitude of these changes was modest and no re-interventions were required during the study period, our findings suggest a potential early impact of RT on valve function. Total and mean heart RT doses, as well as the interval between implantation and RT, were predictive of gradient changes, reinforcing a possible dose-response relationship. Given the short follow-up and small sample size, these results should be interpreted as hypothesis-generating. Longitudinal studies with larger cohorts and detailed dosimetric analyses are needed to fully elucidate the long-term implications of RT on bioprosthetic valve durability and to guide surveillance strategies in this growing patient population.

## Data Availability

The raw data supporting the conclusions of this article will be made available by the authors, without undue reservation.
